# User centered and ontology based information retrieval system for life sciences

**DOI:** 10.1186/1471-2105-13-S1-S4

**Published:** 2012-01-25

**Authors:** Mohameth-François Sy, Sylvie Ranwez, Jacky Montmain, Armelle Regnault, Michel Crampes, Vincent Ranwez

**Affiliations:** 1 LGI2P Research Centre, EMA/Site EERIE, Parc scientifique G. Besse, 30 035 Nîmes cedex 1, France; 2 Inserm/Institut Multi-Organismes, Immunologie, Hématologie et Pneumologie (ITMO IHP), 175, rue du Chevaleret, 75013 Paris, France; 3 Institut des Sciences de l'Evolution de Montpellier (ISE-M), UMR 5554 CNRS Université Montpellier II, place E. Bataillon, CC 064, 34 095 Montpellier cedex 05, France

## Abstract

**Background:**

Because of the increasing number of electronic resources, designing efficient tools to retrieve and exploit them is a major challenge. Some improvements have been offered by semantic Web technologies and applications based on domain ontologies. In life science, for instance, the Gene Ontology is widely exploited in genomic applications and the Medical Subject Headings is the basis of biomedical publications indexation and information retrieval process proposed by PubMed. However current search engines suffer from two main drawbacks: there is limited user interaction with the list of retrieved resources and no explanation for their adequacy to the query is provided. Users may thus be confused by the selection and have no idea on how to adapt their queries so that the results match their expectations.

**Results:**

This paper describes an information retrieval system that relies on domain ontology to widen the set of relevant documents that is retrieved and that uses a graphical rendering of query results to favor user interactions. Semantic proximities between ontology concepts and aggregating models are used to assess documents adequacy with respect to a query. The selection of documents is displayed in a semantic map to provide graphical indications that make explicit to what extent they match the user's query; this man/machine interface favors a more interactive and iterative exploration of data corpus, by facilitating query concepts weighting and visual explanation. We illustrate the benefit of using this information retrieval system on two case studies one of which aiming at collecting human genes related to transcription factors involved in hemopoiesis pathway.

**Conclusions:**

The ontology based information retrieval system described in this paper (OBIRS) is freely available at: http://www.ontotoolkit.mines-ales.fr/ObirsClient/. This environment is a first step towards a user centred application in which the system enlightens relevant information to provide decision help.

## Background

As the number of electronic resources grows it is crucial to profit from powerful tools to index and retrieve documents efficiently. This is particularly true in life sciences where new technologies, such as DNA chips a decade ago and Next Generation Sequencing today, sustain the exponential growth of available resources. Moreover, exploiting published documents and comparing them with related biological data is essential for scientific discovery. Information retrieval (IR), the key functionality of the emerging "semantic Web", is one of the main challenges for the coming years. Ontologies now appear to be a de facto standard of semantic IR systems. By defining key concepts of a domain, they introduce a common vocabulary that facilitates interaction between users and softwares. Meanwhile, by specifying relationships between concepts, they allow semantic inference and enrich the semantic expressiveness for both indexing and querying document corpus.

Though most IR systems rely on ontologies, they often use one of the two following extreme approaches: either they use most of the semantic expressiveness of the ontology and hence require complex query languages that are not really appropriate for non specialists; or they provide very simple query language that almost reduces the ontology to a dictionary of synonyms used in Boolean retrieval models [1]. Another drawback of most IR systems is the lack of expressiveness of their results. In most cases, results are simply proposed as a set of resources with no further explanations concerning the match between the resources and the query. Even when an IR system proposes a list of ranked resources, no explanation is provided with regard to (w.r.t.) this ranking, which means the results are not made explicit. In the absence of any justification concerning the results of IR systems, users may be confused and may not know how to modify their query satisfactorily in an iterative search process.

This paper describes an original alternative. Our ontology based information retrieval system (OBIRS) relies on a domain ontology and on resources that are indexed using its concepts (e.g. genes annotated by concepts of the Gene Ontology or PubMed articles annotated using the MeSH, Medical Subject Headings). To fully benefit of this system, queries have to be expressed using concepts of the same ontology. OBIRS' interface thus provides query formulation assistance through auto-completion and ontology browsing. It estimates the overall relevance of each resource w.r.t. a given query. Such an overall relevance is obtained by aggregating the partial similarity measurements between each concept (that may be weighted) of the query and those indexing the resource. Aggregation operators we use are preference models that capture end user expectations. The retrieved resources are ordered according to their overall scores, so that the most relevant resources (indexed with the exact query concepts) are ranked higher than the least relevant ones (indexed with hypernyms or hyponyms of query concepts). More interestingly, defining an overall adequacy based on partial similarities enables a precise score to be assigned to each resource w.r.t. every concept of the query. We summarize this detailed information in a small explanatory pictogram and use an interactive semantic map to display top ranked resources. Thanks to this approach, the end user can easily tune the aggregation process, identify, at a simple glance, the most relevant resources, recognize entities adequacy w.r.t. each query concept, and identify the most discriminating ones.

The main contribution of this work is to favor interactivity between end users and the information retrieval system (IRS). This interactivity is based on the explanation of how a resource is ranked by the IR system itself: explaining how the relevance of a resource is computed provides additional knowledge that is useful to end users to more appropriately reformulate their query. This is achieved by evaluating how well each resource matches the query based on both query/resource index semantic similarities and end user preferences and by providing a visual representation of retrieved entities and their relatedness relation to each query concept. Note that this visual representation does not aim to represent the large number of documents contained in the database to visually identify related ones - as proposed for instance by [2] for genes indexed by concepts of the Gene Ontology - but to represent a small subset of the most relevant ones with visual indications of their relatedness to the query.

The state of the art below starts by presenting general aspects of IR systems. It details particularly operators that are used to aggregate different query concepts, query expansion and the different approaches of similarity measurement used in this context. Then, the methods section describes a new resource-query matching model based on multi-level aggregation of relevance scores. The results section starts by comparing OBIRS engine with some other methods on a benchmark. Then the interactive query rendering interface of OBIRS is detailed. A case study is carried out that aims at identifying transcription factors involved in hemopoiesis pathway. Synthesis and perspectives of this work are then given in the conclusion section.

### Information retrieval systems overview

The contribution of this paper is related to the use of semantics for information representation and visualization in information retrieval systems.

Information retrieval is generally considered as a subfield of computer science that deals with the representation, storage, and access of information. The field has matured considerably in recent decades because of the increase in computer storage and calculus capacity and the growth of the World Wide Web. Some domains, such as life sciences, have particularly benefited from this technological advance. Nowadays, people no longer labor to gather general information, but rather to locate the exact pieces of information that meet their needs [3,4]. The main goal of an information retrieval system (IRS) can thus be defined as "*finding material (usually documents) that satisfies an information need from within large collections (usually stored on computers)*" [5]. The main use of an IRS can thus be summarized as follows: needing information within an application context, a user submits a query in the hope of retrieving a set of relevant resources. To achieve this goal, IRSs usually implement three processes [6]:

• The indexation process aims at representing resources (often documents) and queries with sets of (weighted) terms (or concepts) that best summarize their information content.

• The search is the core process of an IRS. It contains the system strategy for retrieving documents that match a query. An IRS selects and ranks relevant documents according to a score strategy that is highly dependent on their indexation.

• The query expansion is an intermediate process that reformulates the user query, based on internal system information, to improve the quality of the result.

In most IRSs, the indexation process boils down to representing both documents and queries as a bag of weighted terms (often called keywords) [7]. IRSs that use such document representation are keyword-based. A serious weakness of such systems is that they can be misled by the ambiguity of terms (e.g. homograph) and ignore relationships among terms (e.g. synonym or hypernym) [8]. To overcome this difficulty, recent IRSs map keywords to the concepts they represent [9]. These concept-based IR systems thus need general or domain conceptual structures on which to map the terms. Conceptual structures include dictionaries, thesauri (Wordnet, UMLS) or ontologies (e.g. Gene Ontology). It is now widely acknowledged that their use significantly improves the performance of IRSs [10], and there is still room for improvement since most ontologies are not optimized to achieve this goal [11]. A survey of concept-based IR tools can be found in [9]. Many concept-based IRSs were developed based on theoretical frameworks for the indexing process as well as for relevance measurement [12]. The latter assigns a score to each document (called RSV - *Retrieval Status Value) *depending on how well it matches the query.

**Figure 1:**
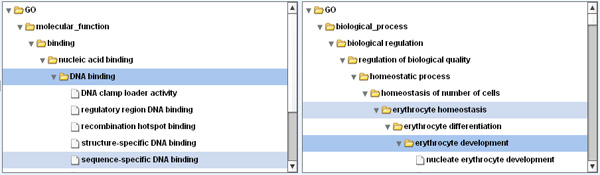
**Exploration of gene ontology concepts hierarchy in OBIRS**. OBIRS allows navigating within the concepts hierarchy to assist the query formulation. The user can be aware that "*sequence-specific DNA-binding*" is a specialisation of "*DNA binding*" and that "*erythrocyte homeostasis*" is a generalisation of "*erythrocyte development*".

The work presented here is in line with the concept-based approach and takes as a starting point the existence of domain ontology. Both resources and queries are represented by a set of concepts from this ontology. Let us see on an example based on the Gene Ontology (GO) how ontologies can help reduce the number of relevant documents missed by Boolean IRSs (i.e. silences). Here resources are genes from the UniProt database, that have been indexed by GO concepts [13]. Such gene indexing were originally done manually using experimental evidence or through sequences' similarities. Note that recent works propose to mine the scientific literature in order to enrich conceptual indexation of genes [14] or to retrieve scientific articles [15-18]. Having the following concepts set as query: {"*erythrocyte development*", "*DNA binding*"}, our system retrieves, among the 30 best results, the gene *HOXB6 *that is relevant though indexed by none of the query concepts. Indeed in its annotation one may find: "*sequence-specific DNA binding*" and "*erythrocyte homeostasis*", the first concept being a specialisation of "*DNA binding*" and the second one being a generalisation of "*erythrocyte development*" (see Figure 1 for excerpt of query concepts tree). Hence a Boolean search engine will not retrieve such gene (no exact match between query and indexation) but by extending query concepts to hyponyms and hypernyms ontology based information retrieval systems do. Such automatic query expansion process have been implemented in PubMed long ago and have been shown to significantly improve document retrieval [19].

### Boolean requests and their generalizations

Boolean requests are certainly the most simple and widespread requests. However, studies indicate that even simple Boolean operators (AND, OR, NOT) are rarely used in web queries [20], and are even sometimes misused [21,22]. Indeed, even when users know that all the terms must be included in the indexation (conjunctive request) or, on the contrary, that only one is needed (disjunctive requests), they do not mention it to the system. In the following, we thus focus on common requests where the user query is only a set of a few concepts.

Minkowski-Hölder's L_p _norms are aggregation operators that provide a theoretical framework to express whether a query is conjunctive or disjunctive using only one parameter [7]. They are particularly well suited to cases where the terms of the request are weighted. These weights may be related to term frequencies within the corpus, e.g. TF-IDF [7], or come from a fuzzy set indexation model. In this latter, a weight is associated with each concept indexing a document to represent to what extent a concept is a reliable indexation of a document [23].

Unfortunately, by summarizing the relevance of the document in a single score, aggregation operators tend to favor information loss and to fuzz out query results [24]. Indeed, unlike end users, they do not differentiate between documents whose scores result from cumulative minor contributions of all concepts within the query and those whose scores are due to the major contribution of a single concept. In addition, as they do not take advantage of semantic resources (ontologies, thesauri), they are unable to find relevant documents that are indexed by concepts that are different but semantically related to those of the query. Indeed, these operators only aggregate weights of a sub-set of terms: the ones that appear in the query. This statement is the basis of query expansion.

### Query expansion

Query expansion is an intermediary step between the indexing and the matching process. As stated in [25], end users can rarely perfectly formulate their needs using query languages because they only have partial knowledge of IRS strategy, of the underlying semantic resources, and of the content of the database. Based on this statement, (semi-)automatic query refinement and expansion strategies have been developed. These reformulations may modify a query by adding concepts to it, by removing "poor" concepts from it or by refining its concepts' weights. Many query expansion techniques have been proposed, among which the widespread *relevance feedback *[26]. This query expansion technique uses the documents that are judged to be relevant by the user after an initial query to produce a new one using reformulation, re-weighting and expansion [27]. When done automatically, this process is called relevance back-propagation [28].

Query expansion may also be based on external vocabulary taken from ontologies or thesauri [29]. A common expansion strategy aims at supplementing the query through adding its concepts' hyponyms. This method is an interesting complement to the Boolean search system detailed above. Indeed, it is then possible to select documents that are not indexed using exactly the same terms as the query and thus avoid *silences*. This strategy is used for instance by the IRSs of PubMed [30] and GoFish [31]. However, since no distinction is made between the initial terms and those added, users may be puzzled by the set of documents retrieved. Indeed, since they are not aware their query has been altered, they may not be able to understand the selection of a document indexed with none of their query terms. Moreover, query expansion can lead to disseminate the most relevant documents within a very long list of results. XploreMed [32], ClusterMed [33] or GoPubMed [16] try to overcome these problems by structuring and filtering search results in a semantic manner while Textpresso relies on predefined semantic categories to refine document search in a given field (e.g neuroscience [34] or site-specific recombinases [17]). We propose an alternative solution where the score of a document vary depending on whether it is indexed by an exact query concept, a semantically close concept or a distant one. This allows our system to identify and retrieve a subset of the most relevant documents and to graphically represent these scoring subtleties to explicit document relevance with respect to the query.

### Semantic similarity measurements

It is possible to improve query expansion by using similarity measures. These measures not only enable selection of documents indexed with terms related to those of the query, but also retrieved documents to be ranked according to their semantic similarity to the query.

Since our approach extensively relies on semantic similarity measurements that significantly impact RSV calculus (*Retrieval Status Value*, see state of the art section), we detail some of them below. As some of these measures satisfy distance axioms, we use semantic proximity, closeness or similarity randomly in the following.

The similarity measurements that have been proposed can be grouped in two main categories depending whether they are defined by intention or by extension. The first use the semantic network of concepts as metric space, and the second use a statistical analysis of term appearance in a corpus of documents [35].

While the semantic network may include various kinds of concept relationships, most intentional similarity measures only rely on the subsumption relationship, denoted as *is-a *[36]. Indeed this relationship is the only one shared by all ontologies and it constitutes their backbone. The key role of the *is-a *relationship is clearly made explicit in the formal definition of an ontology proposed by [37]. The set of *is-a *relationships among concepts can be conveniently represented by a directed graph whose vertices are concepts and whose edges indicate their subsumption relationship (*is-a*). Many concept similarities are based on this *is-a *graph. One of the most straightforward uses of this graph structure is to consider the length of the shortest path between two concepts *C*_*1 *_and *C*_*2 *_as their semantic distance [36]. If all the edges of the path have the same orientation, one concept is subsuming the other, but the more changes in direction the path contains, the harder it is to interpret. Therefore, [38] proposes to adapt this classical graph distance to produce a more sensitive proximity measurement, π_*H0*_(*C*_1_, *C*_2_), which takes into account the length of the path *P *between *C*_*1 *_and *C*_*2*_, *lg*(*P*) and the changes in direction within the path, *nbC*(*P*):

(1) $$\pi_{\text{HO}}\left({\text{C}}_{\text{1}},{\text{C}}_{\text{2}}\right)={\text{min}}_{\text{P = (C1}\to \text{C2)}}\mathrm{lg}\left(\text{P}\right)+\text{K}\;\text{*}\;\text{nbC(P)}$$

The *K *factor modulates the influence of changes in direction on the overall measurement. When K = 0, *π*_*H0 *_is equivalent to the distance proposed in [36]. On the other hand, a high value of K implies a minimum number of changes and thus a path that meets either one of the *least common ancestors *of *C*_*1 *_and *C*_*2*_, denoted by *lca*(*C_1_*, *C*_*2*_) or one of their *greater common descendants*, denoted by *gcd(C_1_, C_2_)*. Since 1994, when [39] first proposed to use *lca *in this context, it has played a key role in several similarity measurements. However, while focusing on the *lca*, this measurement neglects the symmetric notion of *gcd *and completely ignores whether concepts share common descendants, or not.

One main limitation of all these graph-based measurements is that they assume edge homogeneity, whereas each edge of the *is-a *graph represents a specific degree of generalization or specialization. The semantic measurement proposed in [40] tries to capture this information based on the number of descendants of each concept. As this measurement is based on the *is-a *graph, it is denoted *d*_*ISA *_and the authors demonstrate that it satisfies distance axioms. More formally, denoting by *S*_*c *_a set of concepts from an ontology, by *hypo(S*_*c*_*) *the set of concepts that are hyponyms of at least one concept of *S*_*c *_and by *ancEx(C*_*1*_*, C*_*2*_*) *the set of concepts that are ancestors of either *C*_*1 *_or *C*_*2 *_(but not of both), *d*_*ISA *_is defined as:

(2) $${\text{d}}_{\text{ISA}}\left({\text{C}}_{\text{1}}\text{,}{\text{C}}_{\text{2}}\right)=\left|\text{hypo(ancEx(}{\text{C}}_{\text{1}}\text{,}{\text{C}}_{\text{2}}\text{))}\cup \text{hypo(\{}{\text{C}}_{\text{1}}\text{\} )}\cup \text{hypo(\{}{\text{C}}_{\text{2}}\text{\} )}-\text{hypo(\{}{\text{C}}_{\text{1}}\text{\} )}\cap \text{hypo(\{}{\text{C}}_{\text{2}}\text{\} )}\right|$$

In this approach, the information content of a concept is evaluated by *intention *using only the ontology but not the corpus. Alternatively, *Extensional *measurements are mostly based on the corpus and often rely on the concept *information content *(or *IC*) defined in [35]. The *IC *of a concept *C*_*1 *_is derived from the probability *P*(*C*_*1*_) that a document of the corpus is indexed by *C*_*1 *_or one of its descendants:

(3) $$\text{IC(}{\text{C}}_{\text{1}}\text{)}=-\log\;\left(\text{P(}{\text{C}}_{\text{1}}\text{)}\right)$$

Combining the ideas of *lca *and *IC*, [35] introduces the notion of the most informative common ancestor (*MICA*) of a pair of concepts and defines a semantic proximity based on it as: π_Resnik _= IC(MICA(C_1_, C2)). It should however be noted that *MICA(C*_*1*_*, C*_*2*_*) *is not necessarily a *lca *of *C*_*1 *_and *C*_*2*_. This proximity measurement is tightly correlated with the individual *IC *of the two concepts. [41] proposes a variant to correct this bias:

(4) $$\pi_{\text{lin}}\left({\text{C}}_{\text{1}},{\text{C}}_{\text{2}}\right)=\frac{2*\text{IC(MICA(}{\text{C}}_{\text{1}}\text{,}{\text{C}}_{\text{2}}\text{))}}{\text{IC(}{\text{C}}_{\text{1}}\text{)}+\text{IC(}{\text{C}}_{\text{2}}\text{)}}$$

[38] proposes another evaluation of *IC *of a concept. The main idea behind such a formulation of *IC *lies in the assumption that a concept with many hyponyms has a greater probability of being present in a given corpus (related to the considered ontology). Indeed, a concept is considered present in a corpus when at least one of its hyponyms is present. The expressiveness of a concept is thus inversely proportional to the number of its hyponyms. It should be noted that the *IC *value is 0 for the root and 1 for leaves.

(5) $$IC\left(C\right)=1-\frac{\log\left(hypo\left(C\right)+1\right)}{\log\left(\max_{con}\right)}$$

Where *max*_*con *_is the number of concepts in the ontology. From now on, we assume that this *IC *estimation is used to define Lin and Resnik proximities. Proximities can be used in different contexts and their choice strongly depends on final objectives. Adequacy with real concepts' relatedness (i.e. the ones given by experts) must also be taken into account within the measurement choice [42,43]. The following section describes our aggregation model, based on a semantic similarity that leads towards relevance scoring of document with respect to a query.

## Methods

### An original multi-level score aggregation to assess documents' relevance based on semantic proximity

Our work refers to concept-based IRSs. Our Retrieval Status Values (RSVs) are calculated from a similarity measurement between the concepts of an ontology. We propose to break down the RSV computation into a three stage aggregation process. First, we start with a simple and intuitive similarity measure between two concepts of the ontology (stage 1); then, a proximity measure is computed between each concept of the query and a document indexing (stage 2); finally, these measures are combined in the global RSV of the document through an aggregation model (stage 3). The last stage (aggregation) captures and synthesizes the user's preferences and ranks the collection of retrieved documents according to their RSV. The aggregation model enables restitution of the contribution of each query term to the overall relevance of a document. Hence it provides our system with explanatory functionalities that facilitate man-machine interaction and support end users in iterating their query. Furthermore in order to favor user interactions concept proximities must be intuitive (so that the end user can easily interpret them) and rapid to compute (so that the IRS is responsive even in the case of large ontologies).

We estimate the similarity of two concepts based on the Jaccard index between their descendant sets. Two main objectives are followed here: i) avoid silence when no document is indexed with the exact query concepts but with related concepts (hyponyms, hypernyms) to increase the recall of the system; ii) make the query results more explicit concerning the way a match is computed, in particular documents indexed by query concepts and documents indexed by hyponyms or hypernyms need to be distinguished.

### Semantic similarity between concepts and sets of concepts

The choice of the semantic similarity measurement used by our IRS has a major impact on: i) the relevance of the retrieved documents, ii) the system's recall and iii) user comprehension of the document selection strategy. Hence, we propose a variant of the similarity measurement proposed by [41], with a valuation of the informational content of a concept based on the number of its hyponyms [44].

Because it has been emphasized that query concepts should only be replaced by hyponyms or hypernyms, we estimate the semantic proximity of two concepts based on how much their hyponyms overlap (using the Jaccard index) as long as one is a hyponym of the other and otherwise we fix it at 0:

(6) $$\pi_{\text{JD}}\left({\text{C}}_{\text{1}},{\text{C}}_{\text{2}}\right)=\left\{\begin{array}{cc} \frac{\left|\text{hypo(\{}{\text{C}}_{\text{1}}\text{\} )}\cap \text{hypo(\{}{\text{C}}_{\text{2}}\text{\} )}\right|}{\left|\text{hypo(\{}{\text{C}}_{\text{1}}\text{\} )}\cup \text{hypo(\{}{\text{C}}_{\text{2}}\text{\} )}\right|} & \text{if}\;{\text{C}}_{\text{1}}\in \text{hypo(\{}{\text{C}}_{\text{2}}\text{\} )}\;\text{or}\;{\text{C}}_{\text{2}}\in \text{hypo(\{}{\text{C}}_{\text{1}}\text{\} )}\\ \text{0} & \text{otherwise} \end{array}\right)$$

It should be noted that π_JD_(C_1_, C_2_) is comprised between 0 and 1. π_JD_(C_1_, C_2_) = 0 if, and only if, *C*_*1 *_and *C*_*2 *_have no hyponym relationship while π_JD_(C_1_, C_2_) = 1 if, and only if, C_1 _= C_2 _(same concept).

Several solutions have been proposed to extend similarity measurement between two concepts to measurement of similarity between two sets of concepts. This problem is of particular interest in life sciences because similarity between two gene indexations through the Gene Ontology (GO) may provide hints on how to predict gene functions or protein interactions [45]. Whereas comparing gene indexations (and document indexing in general) requires similarity measurements to be symmetric, this is not the case in IR. Indeed, when matching documents to queries, it seems normal to penalize a document because one concept of the query is absent from its indexing; on the other hand, penalizing a document because it is indexed by one concept absent from the query would be rather odd. This latter remark leads to define the proximity between an elementary query (made of a single concept) and a document as the maximum value of the similarities calculated between the query concept and each concept of the document indexing. By extension, this leads to a simple and intuitive proximity measurement between each query concept and a document based on the maximum operator. More formally, if π denotes the similarity between two concepts from an ontology *O*, and *D*_*i *_denotes the *i*^*th *^concept of document *D *index, i = 1..|D|, then we define the similarity between a concept Q_t _of the query and *D *as π(Q_t_, D) max_0≤i≤|D| _π(Q_t_, D_i_).

### Proximity measurement between a document and a query

After determining similarities between each concept of the query and (the index of) a document, the next step consists in combining them in a single score that reflects the global relevance of the document w.r.t. the query. User's preferences have to be taken into account during this process in order to determine the overall relevance of a document w.r.t. a query, i.e. its RSV.

As mentioned above, computing documents' RSV enables them to be ranked according to their relevance. Furthermore having the score details of a document for each query concept allows us to justify and compare the source of the match of each document with the query. This is clearly related to the preference representation problem that has been extensively studied in decision theory [46]. A classical solution is to define a utility function *U *in such a way that, for each alternative **D**, *D*' in a list **D** of alternatives, **D**≽ *D*' (i.e. **D** is preferred to *D'*) if *U*(**D**) ≥ *U*(*D*'). The decomposable model of Krantz [47] has been widely used when alternatives are n dimensional. Following this model the utility function *U *is defined as: U(q_1_,..,q_n_) = h(u_1_(q_1_),..,u_n_(q_n_)) where u_t_(. ), t = 1.. n, are real-valued functions in [0, 1] and h: [0, 1]^*n *^→ [0, 1] is an aggregation operator that satisfies the following conditions:

• h is continuous;

• h(0, 0,..., 0) = 0 and h(1, 1,..., 1) = 1;

• h is monotonous: ∀*j in 1..n *if *a*_*j *_≥ *b*_*j *_then h(a_1_,...,a_*n*_) ≥ h(b_1_,...,b_*n*_)

In our context, the n dimensional space corresponds to n query concepts. The n coordinates of a document correspond to its proximities with each concept of the query, i.e., π(Q_t_, D), t = 1.. n, defined in the previous section correspond to the u_t_(.) functions. The aggregation model combines the degrees of relevance (or matches) of a document indexing w.r.t. each query concept w.r.t. the user's preferences. The aggregation function *h *captures the preferences of the user: the way the elementary degrees of relevance are aggregated depends on the role of each query term w.r.t. the user's requirements. Three kind of aggregation can be distinguished:

• conjunctions (AND), $h\left(\pi \left(Q_{1},D\right),..,\pi \left(Q_{\left|Q\right|},D\right)\right)\leq \underset{t=1..|Q|}{\min}\pi \left(Q_{t},D\right)$;

• disjunctions (OR), $h\left(\pi \left(Q_{1},D\right),..,\pi \left(Q_{\left|Q\right|},D\right)\right)\geq \underset{t=1..|Q|}{\max}\pi \left(Q_{t},D\right)$;

• compromises, $\underset{t=1..|Q|}{\min}\pi \left(Q_{t},D\right)\leq h\left(\pi \left(Q_{1},D\right),..,\pi \left(Q_{\left|Q\right|},D\right)\right)\leq \underset{t=1..|Q|}{\max}\pi \left(Q_{t},D\right)$.

With the goal of improving man/machine interaction, we hope to give users a friendly and intuitive way of expressing their preferences concerning the overall relevance scoring strategy between a document and a query. We thus focus on compromise operators because they fit the widespread decision strategy that constrains the overall score to be between the minimum and the maximum value of elementary scores (convexity). Our approach is consequently based on Yager's operators [48]. These define a parameterized family of functions that represents compromise operators:

(7) $$Y_{m}\left(\pi \left(Q_{1},D\right),..,\pi \left(Q_{\left|Q\right|},D\right)\right)={\left(\left(\overset{\left|Q\right|}{\underset{t=1}{\sum }}\pi {\left(Q_{t},D\right)}^{q}\right)∕|Q|\right)}^{1∕q},q\in \mathbb{R}$$

To get a better idea of the wide range of aggregation functions that are possible with this operators' family, let us exert some remarkable values:

• q = 1, arithmetic mean,

• q = -1, harmonic mean,

• q → 0, geometrical mean,

• q → + &#8734, max(OR generalization)

• q → - &#8734, min (AND generalization)

A compromise operator can thus be selected by the user who may simply provide the value of parameter q. The choice of an aggregation operator is simply reduced to the choice of parameter q which still corresponds to our intuitive man/machine requirements. Indeed, our IRS interface includes a cursor to control the value of parameter q and to indicate whether the aggregation should tend toward a generalized "or", a generalized "and", or should tolerate more or less compensatory effects.

When criteria do not play a symmetric role in the aggregation process, the relative importance of criteria can also be introduced in aggregation operators. In our case, it is possible to check that the Yager family can be extended to the weighted operators' family:

(8) $${Ȳ}_{wm}\left(\pi \left(Q_{1},D\right),...,\pi \left(Q_{\left|Q\right|},D\right)\right)={\left(\overset{\left|Q\right|}{\underset{t=1}{\sum }}p_{t}.\pi {\left(Q_{t},D\right)}^{q}\right)}^{1\;/)\;q},\overset{\left|Q\right|}{\underset{t=1}{\sum }}p_{t}=1$$

When the above weighted operators's family is used, the user has to fit both q parameter and the weights distribution upon the query terms. In order to keep the query terms weights selection simple and intuitive, our IRS interface allows the user to move cursors (one by query terms) and to see inline effects of that change in results.

This RSV 3-step computation (i.e. concept/concept, concept/document, query/document) has been integrated in an efficient and interactive querying system as detailed in the following section. Note that this 3-step strategy can be used with any concept/concept similarity measure. Our querying system, OBIRS, let the user chose between the Lin (selected by default) and the Jaccard proximities.

## Results

Querying systems endowed with query expansion that add hyponym concepts to the query can be seen as the first step towards a semantic querying system. Our approach refines basic solutions to avoid silences by selecting documents that are indexed by the semantically closest hyponyms or hypernyms of the query concepts. Furthermore, we are convinced that users should easily be able to understand the RSV at a glance to favor interaction with the IRS during query reformulation. Our 3-stage relevance model (which allows RSVs to be computed) integrates both the semantic expressiveness of the ontology based data structure and the end-user's preferences. The more user friendly the man-machine interface, the more efficient the interaction between the IRS and the end-user.

The 3-step relevance model presented in this paper has been implemented and a web-based client is available through [49]. The model is experimentally validated as follows. First we perform experiments to determine the impact of similarity measurement using the MuchMore collection [50] and secondly we use OBIRS in a use case dedicated to gene identification.

### OBIRS results on an experimental campaign

To study the impact of *IC *based semantic similarity measures on OBIRS' performances, we need to fix system parameters such as *q *value (set to 2.0), number of retrieved documents (1,000) and *RSV *threshold (0.0, i.e. no filtering). Three measures have been implemented and used for this experiment: Lin, Resnik and Jaccard. Our search strategy is also compared with Boolean search using AND/OR operators.

The MuchMore collection consists of 7,823 medical paper abstracts and 25 queries with their relevance judgments. Documents and queries in that collection are indexed using MeSH concepts. The evaluation methodology used for this campaign follows the TREC protocol [51]. Note that during experiment, some query terms and document terms haven't been mapped to MeSH concepts leading to smaller precision values than expected. This issue is known as semantic coverage problem and is still under analysis.

**Figure 2:**
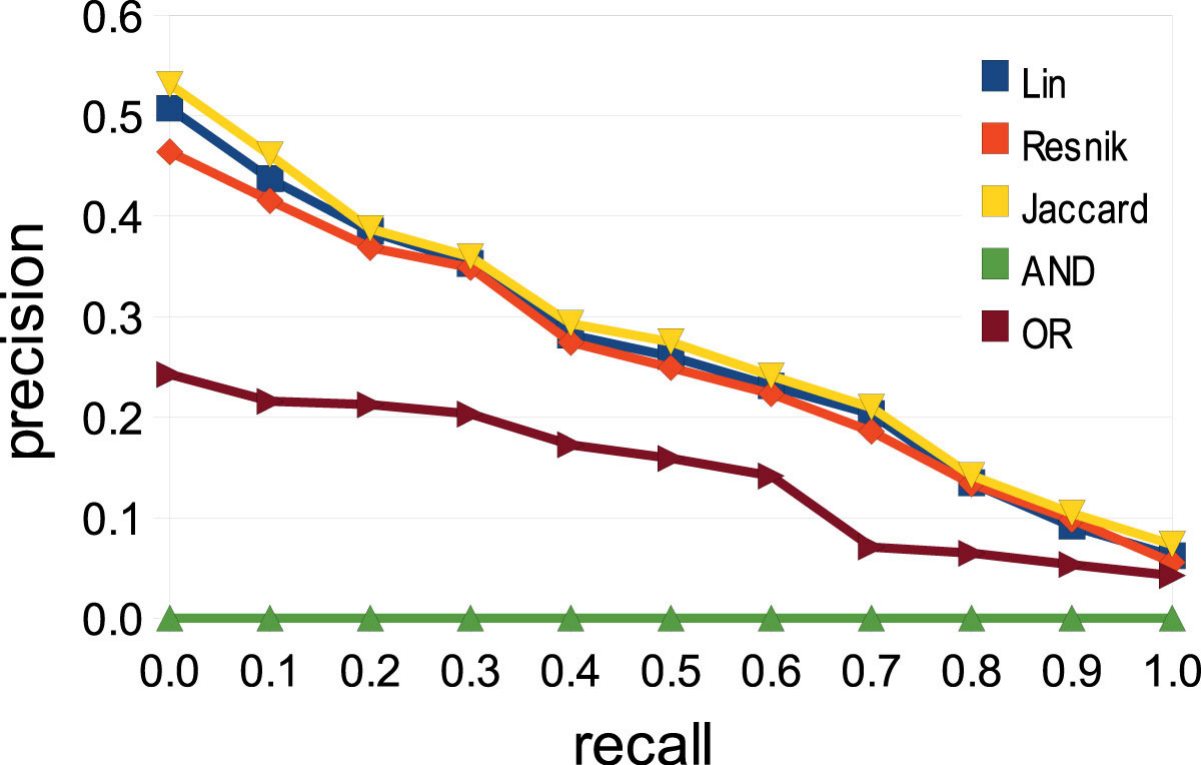
**Interpolated precision-recall curve**. Impact of IC based semantic similarity measures on precision. Five information retrieval approaches are compared using the MuchMore benchmark. Three rely on OBIRS 3-step strategy based on different concept-concept semantic proximities (Lin, Resnik and Jaccard) and the two others are Boolean search based on AND/OR operators.

Results are summarized in Figure 2 by the variation curve of the system precision for ten recall points (interpolated precision-recall curve). OBIRS performances with Lin, Jaccard or Resnik proximities are comparable and far better than those obtained by a basic Boolean search using AND or OR operators. The online version thus let the user choose between the Jaccard proximity and the Lin one that is semantically richer but also harder to interpret.

Before detailing case studies it is necessary to describe OBIRS user interface and main functionalities.

### Overview of OBIRS user interface

**Figure 3:**
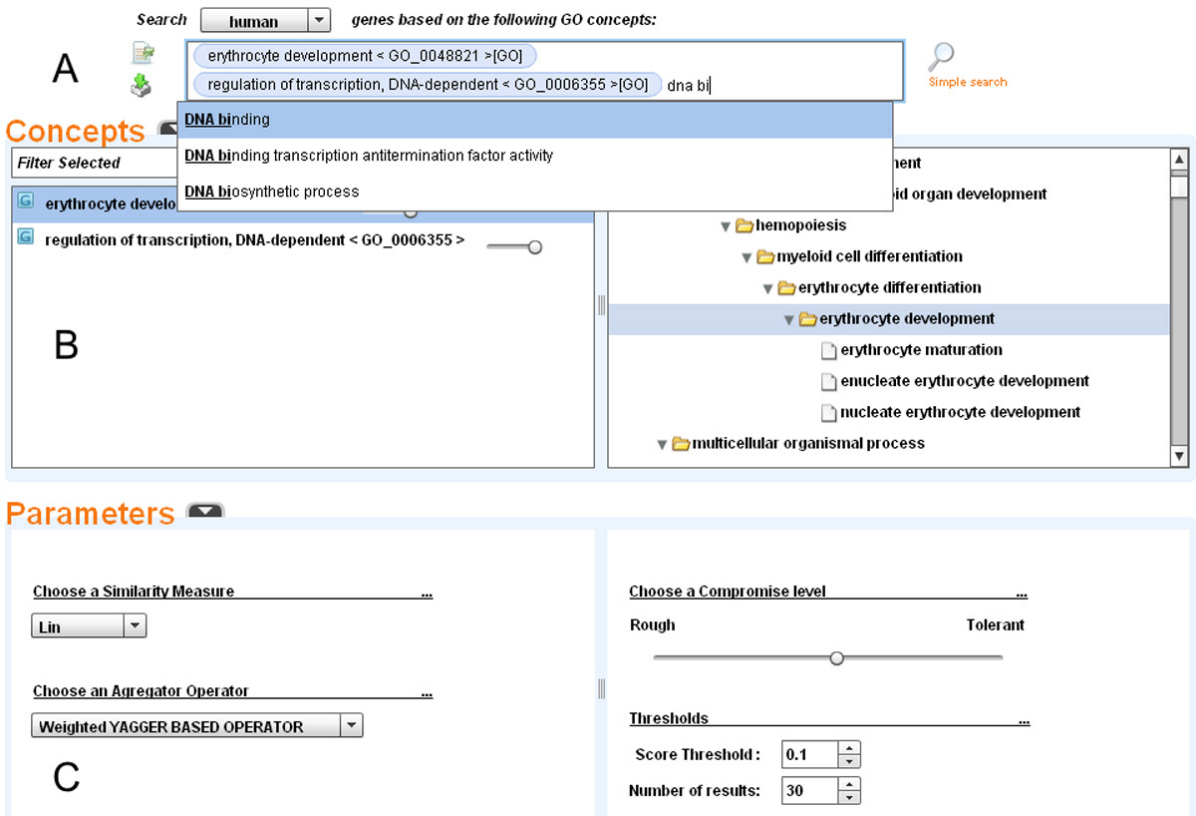
**OBIRS querying interface**. **A**. Input with auto-completion functionality. **B**. Visualisation of query concepts' position within the Gene ontology. **C**. Parameters panel setting.

The screenshot presented in Figure 3 shows an overview of OBIRS querying website interface. The loaded corpus contains the whole genome of 6 species (*Homo sapiens*, *Mus musculus*, *Plasmodium falciparum*, *Danio rerio, Oryza sativa, Arabidopsis thaliana*). The querying field of this website (Figure 3-A) allows users to retrieve genes, of a given species, that are related to some GO concepts [49]. Auto-completion assistance is provided to help users to set query GO concepts. Using the *advance search *link, users may see for each selected concept of the query its position within the GO hierarchy (Figure 3-B). They may also adjust each concept weight to give more influence to certain concepts. Figure 3-C shows the parameters' setting panel, where users can easily tune the aggregation function according to their preferences by moving a cursor from *rough *(strict conjunctive - "AND") to tolerant (disjunctive - "OR"), limit the number of retrieved documents (here 20) and fix a threshold for the RSV (here 0.1).

**Figure 4:**
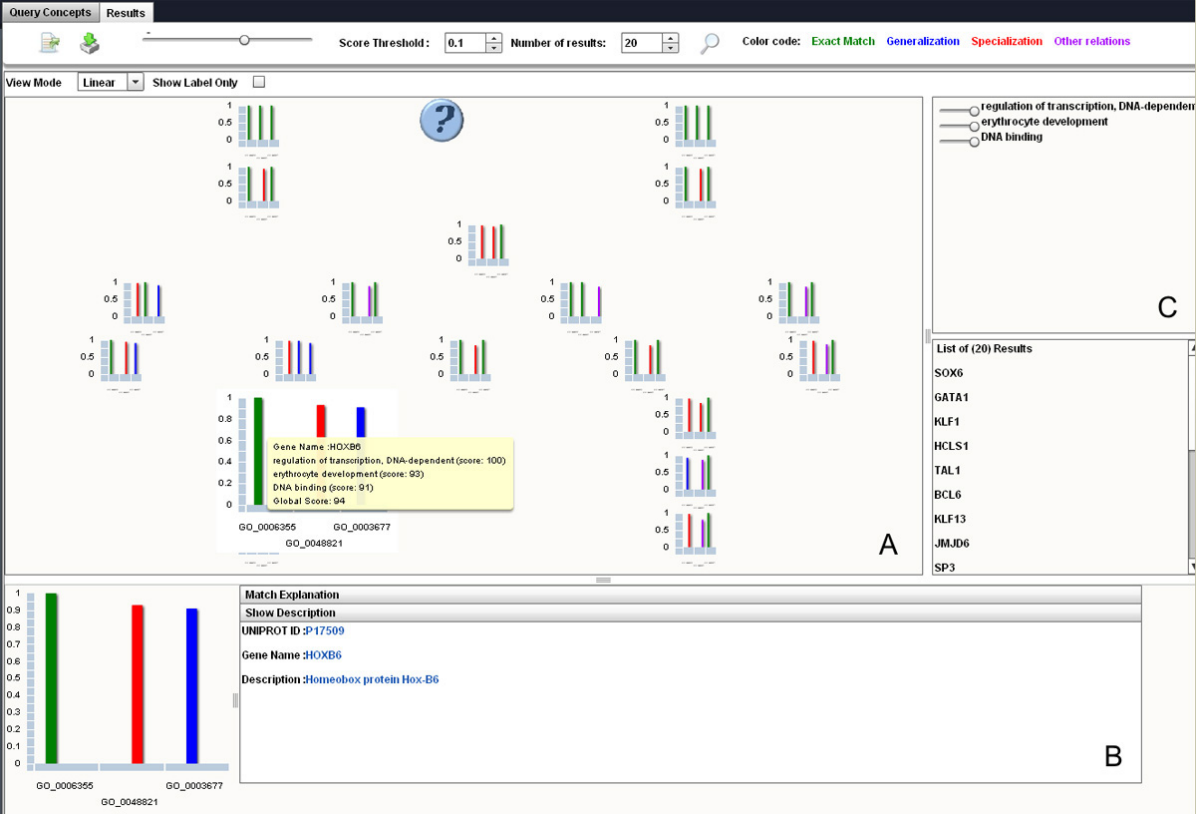
**OBIRS result interface**. Genes returned by OBIRS while querying with {"*erythrocyte development*", "*regulation of transcription*, *DNA*-*dependent*", "*DNA binding*"} are displayed on a semantic map according to their relevance (**4-A**). When selecting a gene on this map (here *HOXB6*) detailed information are provided (**4-B**). The user can move cursors to adjust concept weights for query reformulation (**4-C**).

Once the (parameterized) query is completed, results appear on another screen (Figure 4). The IRS selects relevant genes and displays them on a semantic map (Figure 4-A) in such a way that their physical distance to the query symbol (blue circle with question mark in the middle of the screen) is proportional to the RSV values. Each gene may be displayed either by a pictogram or by its official symbol (the *show label only *option). Users can thus identify at a glance the most relevant genes. The pictogram details adequacy between gene annotations and the query: the contribution of each query concept to the RSV assessment is synthesized in a histogram where a bar is associated with each concept *Q*_*t *_of the query. This bar is coloured depending on whether the closest (according to the chosen semantic similarity measure) concept of the gene annotation is exactly *Q*_*t *_(green), a hyponym (red) or a hypernym (blue) of *Q*_*t*_. The bar is purple in other cases. The size of the bar associated with *Q*_*t *_is proportional to the elementary relevance of the document w.r.t. *Q*_*t *_(i.e. *π*(*Q*_*t*_, *D*)). A visual lens synthesizes information of a gene when the mouse hover its pictogram (here *HOXB6*). Further details may be obtained by clicking on it (Figure 4-B): "Show description" gives its official symbol, link towards UniProt database and its description according to UniProt. "*Match explanation*" details each query concept's elementary relevance.

To refine their queries, users can change relative importance of query concepts by adjusting their weight (Figure 4-C). Modifying a weight refreshes the visualisation screen and histogram positions change in order to take into account new weight values. Results may be exported as CSV or XML.

It should be noted that expanding query with hyponyms and hypernyms *de facto *increases *recall *and decreases the *precision *of an IRS. However in OBIRS, since users may distinguish at a glance the most relevant genes, they benefit of query expansion without its downside.

### Cases studies: application to gene identification

This section describes two case studies illustrating the relevance of OBIRS for gene retrieval.

During the generation of red blood cells which is called the erythropoiesis, the expression of several transcription factors is required in progenitor cells to induce their differentiation. Amongst these genes, some such as *GATA1, TAL1 *and *SP3 *are known to be essential. Here OBIRS is used in order to obtain the list of known transcription factors involved in human *hemopoiesis *pathway. Our query was made of three concepts: {"*erythrocyte development*", "*regulation of transcription, DNA-dependent*", "*DNA binding*"} limiting result to the best 30 genes (those with highest score). The first 30 genes were known genes amongst which 22 were linked to erythropoiesis and the remaining ones were involved either in leukemia derived from red blood cell precursors or in more embryonic steps of blood formation. Moreover, the top 15 genes were of strong interest (amongst them *SP3, GATA1*, *TAL1*). Despite the large number of human genes in UniProt database (~45.000 genes) and GO concepts (about 30.000 concepts) the result of this query is obtained in a few seconds. The second case study is focused on *Zebrafish*, a model organism used in agronomy to study fish immune responses to viruses (because its genome is simple, fully sequenced and well annotated). During viral infections, many genes are involved in the anti-viral response, amongst which, those responsible for the inflammation. However, the inflammation can also be induced by other conditions such as autoimmune diseases or cancers. Here OBIRS is used in order to obtain the list of known genes involved in this anti-viral response. Our first query was made of two (un-weighted) concepts: {"*defense response to virus*" and "*inflammatory response*"} limiting result to the best 20 genes. Most of the retrieved genes were of strong interest however some, such as the gene *PXK*, were not directly related to anti-viral response but to lupus, an autoimmune condition which induces also inflammation [52]. We thus refined our query by giving more weight to "*defense response to virus*" as compare to "*inflammatory response*" (100 vs 50). The new result contained 19 viral-reponse related genes plus a locus (LOC565099) having no gene name. As expected, *PXK *is no longer in the top result list.

## Conclusions

The approach described in this paper is an important step towards an IRS that benefits from the semantic expressiveness of ontologies while remaining easy to use. An original three stage aggregation model has been described to compute RSV scoring. This model has the particularity to embed end user preferences. The resulting OBIRS prototype is one of the first IRS able to elucidate its document selection to the user thanks to the decomposition of the RSV score that can be transcribed through intuitive pictograms. By locating these pictograms on a semantic map, OBIRS provides an informative overview of the result of the query and new possible interactions. We are currently working on an OBIRS extension that will let users reformulate their query by graphically selecting the documents they value and those in which they have no interest. This reformulation can be done by adding/removing concepts from the query, specifying/generalizing initial concepts of the query or adjusting the aggregation function. Reformulation leads to several optimization and mathematical questions but also raises important issues concerning feedback to users to enable them to continue to understand the IRS process and fruitfully interact with it. We believe that there are many advantages to coupling the IR engine and rendering the result of the query, and that they should be considered simultaneously to provide a new efficient, interactive query environment. The RSV decomposition described in this paper is a good example of the benefit of simultaneously considering two related problems: i) how to rate documents w.r.t. a query ii) how to provide users feedback concerning rating of the documents. The latter is crucial to favor user/IRS intuitive interaction in iterative improvement of the query.

## List of abbreviations

CSV: comma separated values; DNA: Deoxyribonucleic acid; gcd: greater common descendant; GO: gene ontology; IC: information content; IR(S): information retrieval (system); lca: least common ancestor; MeSH: medical subject headings; MICA: most informative common ancestor; OBIRS: ontology based information retrieval system; RSV: retrieval status value; TF-IDF: term frequency-inverse document frequency; TREC: text retrieval conference; UMLS: Unified Medical Language System; XML: extensible markup language.

## Competing interests

The authors declare that they have no competing interests.

## Authors' contributions

VR and SR initiated and coordinated this project. JM proposed an aggregation model based on decision theory. MC initiated, designed and developed the visualisation API used in OBIRS. Supervised by VR, SR, JM and MC, MFS conceived and developed OBIRS and carried out the Muchmore benchmark evaluation. AR carried out the case study. VR, SR and MFS wrote most of the manuscript, all authors read and approved the final version.

## References

[B1] Vallet D Fernandez M Castells P An ontology-based information retrieval model Proceedings of the 2nd European Semantic Web Conference (ESWC 2005), Volume 3532 of Lecture Notes in Computer Science 2005 Springer Verlag 103 110

[B2] Peltonen J Aidos H Gehlenborg N Brazma A Kaski S An information retrieval perspective on visualization of gene expression data with ontological annotation IEEE International Conference on Acoustics, Speech, and Signal Processing 2010 2178 2181

[B3] Bawden D The dark side of information: overload, anxiety and other paradoxes and pathologies Journal of Information Science 2009 35 2 180 191

[B4] Nelson MR We have the information you want, but getting it will cost you!: held hostage by information overload Crossroads - Special issue on the Internet 1994 1 1 11 15

[B5] Christopher DM Prabhakar R Hinrich S Introduction to Information Retrieval 2008 Cambridge University Press

[B6] Belkin N Ingwersen P Pejtersen AM15th annual international ACM SIGIR conference on Research and development in information retrieval. June 21-24 1992; Copenhagen, Denmark 1992 ACM 352

[B7] Salton G McGill MJ Introduction to Modern Information Retrieval 1986 McGraw-Hill, Inc

[B8] Baziz M Boughanem M Pasi G Prade H Montseny E, Sobrevilla P A fuzzy set approach to concept-based information retrieval Proceedings of the 4th Conference of the European Society for Fuzzy Logic and Technology and the 11e Rencontres Francophones sur la Logique Floue et ses Applications (Eusflat-LFA 2005 joint Conferences): 7-9 september 2005; Barcelona, Spain 2005 Universidad Polytecnica de Catalunya 1287 1292

[B9] Haav HM Lubi TL Caplinskas A, Eder J A survey of concept-based information retrieval tools on the web Proceedings of the 5th East-European Conference, ADBIS 2001: 25-28 September 2001; Vilnius, Lithuania 2001 Technika 29 41

[B10] Andreasen T Nilsson JF Thomsen HE Ontology-based Querying Proceedings of the 4th International Conference on Flexible Query Answering Systems, FQAS'00: 25-28 October 2000; Warsaw, Poland 2000 Larsen HL: Physica-Verlag, Springer 15 26

[B11] Jimeno-Yepes A Berlanga-Llavori R Rebholz-Schuhmann D Ontology refinement for improved information retrieval Information Processing & Management 2010 46 4 426 435 10.1016/j.ipm.2009.05.008

[B12] Van Rijsbergen CJ Information Retrieval 1979 Butterworth-Heinemann

[B13] Barrell D Dimmer E Huntley RP Binns D O'Donovan C Apweiler R The GOA database in 2009-an integrated Gene Ontology annotation resource Nucleic Acids Res 2009 37 Database D396 403 18957448 10.1093/nar/gkn803 PMC2686469

[B14] Plake C Royer L Winnenburg R Hakenberg J Schroeder M GoGene: gene annotation in the fast lane Nucleic Acids Res 2009 37 Web Server W300 304 19465383 10.1093/nar/gkp429 PMC2703922

[B15] Muller HM Kenny EE Sternberg PW Textpresso: an ontology-based information retrieval and extraction system for biological literature PLoS Biol 2004 2 11 e309 10.1371/journal.pbio.0020309 15383839 PMC517822

[B16] Doms A Schroeder M GoPubMed: exploring PubMed with the Gene Ontology Nucleic Acids Res 2005 33 Web Server W783 786 15980585 10.1093/nar/gki470 PMC1160231

[B17] Urbanski WM Condie BG Textpresso site-specific recombinases: a text-mining server for the recombinase literature including Cre mice and conditional alleles Genesis 2009 47 12 842 846 19882667 10.1002/dvg.20575 PMC4963979

[B18] Delfs R Doms A Kozlenkov A Schroeder M Giegerich R, Stoye J GoPubMed: ontology-based literature search applied to GeneOntology and PubMed Proceedings of the German Conference on Bioinformatics 2004, (GCB 2004) October 4-6 2004; Bielefeld, Germany 2004 Springer 169 178

[B19] Lu Z Kim W Wilbur WJ Evaluation of Query expansion using MeSH in PubMed Inf Retr Boston 2009 12 1 69 80 19774223 10.1007/s10791-008-9074-8 PMC2747526

[B20] Jansen BJ Spink A Saracevic T Real life, real users, and real needs: a study and analysis of user queries on the web Information Processing & Management 2000 36 2 207 227 10.1016/S0306-4573(99)00056-4

[B21] Jansen BJ The effect of query complexity on Web searching results Inf Res 2000 6 1 Paper87

[B22] Lucas W Topi H Training for Web search: Will it get you in shape? Journal of the American Society for Information Science and Technology 2004 55 13 1183 1198 10.1002/asi.20074

[B23] Detyniecki M Larsen HL Browsing a video with simple constrained queries over fuzzy annotations Proceedings of the 4th International Conference on Flexible Query Answering Systems, FQAS'00: 25-28 October 2000; Warsaw, Poland 2000 Physica-Verlag, Springer 282 288

[B24] Schamber L Relevance and information behavior Annual Review of Information Science and Technology (ARIST) 1994 29 3 48

[B25] Song M Song IY Hu XH Allen RB Integration of association rules and ontologies for semantic query expansion Data & Knowledge Engineering 2007 63 1 63 75 10.1016/j.datak.2006.10.010

[B26] Crouch CJ Yang B Bekin NJ, Ingwersen P, Pejtersen, AM Experiments in automatic statistical thesaurus construction Proceedings of 15th annual international ACM SIGIR conference on Research and development in information retrieval: June 21-24 1992; Copenhagen, Denmark 1992 ACM 77 88

[B27] Abdelali A Cowie J Soliman HS Improving query precision using semantic expansion Information Processing & Management 2007 43 3 705 716 10.1016/j.ipm.2006.06.007

[B28] Boughanem M Chrisment C Soule-Dupuy C Query modification based on relevance back-propagation in an ad hoc environment Information Processing & Management 1999 35 2 121 139 10.1016/S0306-4573(99)00008-4

[B29] Andreasen T An approach to knowledge-based query evaluation Fuzzy Sets and Systems 2003 140 1 75 91 10.1016/S0165-0114(03)00028-9

[B30] PubMed http://www.ncbi.nlm.nih.gov/pubmed/

[B31] Berriz GF White JV King OD Roth FP GoFish finds genes with combinations of Gene Ontology attributes Bioinformatics 2003 19 6 788 789 10.1093/bioinformatics/btg088 12691998

[B32] Perez-Iratxeta C Bork P Andrade MA XplorMed: a tool for exploring MEDLINE abstracts Trends Biochem Sci 2001 26 9 573 575 10.1016/S0968-0004(01)01926-0 11551795

[B33] ClusterMed http://www.xmarks.com/site/demos.vivisimo.com/clustermed

[B34] Muller HM Rangarajan A Teal TK Sternberg PW Textpresso for neuroscience: searching the full text of thousands of neuroscience research papers Neuroinformatics 2008 6 3 195 204 10.1007/s12021-008-9031-0 18949581 PMC2666735

[B35] Resnik P Semantic similarity in a taxonomy: an information-based measure and its application to problems of ambiguity in natural language Journal of Artificial Intelligence Research 1999 11 95 130

[B36] Rada R Mili H Bicknell E Blettner M Development and application of a metric on semantic nets IEEE Transactions on Systems, Man, and Cybernetics 1989 19 1 17 30 10.1109/21.24528

[B37] Maedche A Staab S Ontology learning for the Semantic Web IEEE Intelligent Systems & Their Applications 2001 16 2 72 79 10.1109/5254.920602

[B38] Hirst G St Onge D Fellbaum C Lexical Chains as representation of context for the detection and correction malapropisms WordNet: An Electronic Lexical Database and some of its applications (Language, Speech, and Communication) 1998 Cambrige, MA, USA: The MIT Press 305 332

[B39] Wu Z Palmer M Verbs semantics and lexical selection Proceedings of the 32nd annual meeting on Association for Computational Linguistics: 27-30 June 1994; Las Cruces, New Mexico 1994 Association for Computational Linguistics: Morgan Kaufmann Publishers 133 138

[B40] Ranwez S Ranwez V Villerd J Crampes M Ontological distance measures for information visualisation on conceptual maps Proceedings of the On the Move to Meaningful Internet Systems 2006: OTM 2006 Workshops, Volume 4278 of Lecture Notes in Computer Science 2006 Springer Berlin/Heidelberg 1050 1061

[B41] Lin D Shavlik JW An information-theoretic definition of similarity Proceedings of the Fifteenth International Conference on Machine Learning: 24-27 July 1998; Madison, Wisconsin, USA 1998 Morgan Kaufmann Publishers Inc 296 304

[B42] Lee WN Shah N Sundlass K Musen M Comparison of ontology-based semantic-similarity measures AMIA Annu Symp Proc 2008 384 388 18999312 PMC2655943

[B43] Pakhomov SV Pedersen T McInnes B Melton GB Ruggieri A Chute CG Towards a framework for developing semantic relatedness reference standards J Biomed Inform 2010 44 2 251 265 21044697 10.1016/j.jbi.2010.10.004 PMC3063326

[B44] Seco N Veale T Hayes J Ramon LDM, Lorenza S An intrinsic information content metric for semantic similarity in WordNet Proceedings of the 16th European Conference on Artificial Intelligence (ECAI 2004): 22-27 August 2004; Valencia, Spain 2004 IOS Press 1089 1090

[B45] Pesquita C Faria D Falcao AO Lord P Couto FM Semantic similarity in biomedical ontologies PLoS Comput Biol 2009 5 7 e1000443 10.1371/journal.pcbi.1000443 19649320 PMC2712090

[B46] Modave F Grabisch M EDK, Paris Preference representation by a Choquet integral: Commensurability hypothesis Proceedings of the 7th International Conference on Information Processing and Management of Uncertainty in Knowledge-Based Systems (IPMU'98): 6-10 July 1998; Paris, France 1998 164 171

[B47] Krantz DH Luce RD Suppes P Tversky A Foundations of measurement, vol. 1: Additive and polynomial representations 1971 Academic Press, New York

[B48] Yager RR Possibilistic decision making IEEE Trans on Systems, Man and Cybernetics 1979 9 388 392

[B49] OBIRS http://www.ontotoolkit.mines-ales.fr/ObirsClient/

[B50] Muchmore http://muchmore.dfki.de

[B51] Voorhees EM Croft WB, Van Rijsbergen K Query expansion using lexical-semantic relations Proceedings of the 17th annual international ACM SIGIR conference on Research and development in information retrieval. 03-06 July 1994; Dublin, Ireland 1994 Springer-Verlag, New York, Inc 61 69

[B52] Harley JB Alarcon-Riquelme ME Criswell LA Jacob CO Kimberly RP Moser KL Tsao BP Vyse TJ Langefeld CD Nath SK Genome-wide association scan in women with systemic lupus erythematosus identifies susceptibility variants in ITGAM, PXK, KIAA1542 and other loci Nat Genet 2008 40 2 204 210 10.1038/ng.81 18204446 PMC3712260

